# Daily Resting Heart Rate Variability in Adolescent Swimmers during 11 Weeks of Training

**DOI:** 10.3390/ijerph17062097

**Published:** 2020-03-22

**Authors:** Sigitas Kamandulis, Antanas Juodsnukis, Jurate Stanislovaitiene, Ilona Judita Zuoziene, Andrius Bogdelis, Mantas Mickevicius, Nerijus Eimantas, Audrius Snieckus, Bjørn Harald Olstad, Tomas Venckunas

**Affiliations:** 1 Institute of Sports Science and Innovation, Lithuanian Sports University, 44221 Kaunas, Lithuania; sigitas.kamandulis@lsu.lt (S.K.); antanas.juodsnukis@lsu.lt (A.J.); jurate.stanislovaitiene@lsu.lt (J.S.); ilona.zuoziene@lsu.lt (I.J.Z.); andrius.bogdelis@gmail.com (A.B.); mantas.mickevicius@lsu.lt (M.M.); nerijus.eimantas@lsu.lt (N.E.); tomas.venckunas@lsu.lt (T.V.); 2 Institute of Physical Performance, Norwegian School of Sport Sciences, 0863 Oslo, Norway; b.h.olstad@nih.no

**Keywords:** autonomic nervous system, competitive swimming, high-intensity exercise, sleep, training volume

## Abstract

Adolescent athletes are particularly vulnerable to stress. The current study aimed to monitor one of the most popular and accessible stress markers, heart rate variability (HRV), and its associations with training load and sleep duration in young swimmers during an 11-week training period to evaluate its relevance as a tool for monitoring overtraining. National-level swimmers (n = 22, age 14.3 ± 1.0 years) of sprint and middle distance events followed individually structured training programs prescribed by their swimming coach with the main intention of preparing for the national championships. HRV after awakening, during sleep and training were recorded daily. There was a consistent ~4.5% reduction in HRV after 3–5 consecutive days of high (>6 km/day) swimming volume, and an inverse relationship of HRV with large (>7.0 km/day) shifts in total training load (r = −0.35, *p* < 0.05). Day-to-day HRV did not significantly correlate with training volume or sleep duration. Taken together, these findings suggest that the value of HRV fluctuations in estimating the balance between the magnitude of a young athlete’s physical load and their tolerance is limited on a day-to-day basis, while under sharply increased or extended training load the lower HRV becomes an important indicator of potential overtraining.

## 1. Introduction

For training to be effective, it is essential to have both appropriate planning and to implement a training load monitoring and adjustment system that includes biofeedback [1]. Sport practitioners are constantly looking for ways to most objectively, quickly, and cost-effectively design individualized training programs. Such programs are based on the individual adaptation, as reflected by the recuperation (restitution) and super-compensation of the body functions during the recovery period after training sessions, to maximize long-term gains in performance while at the same time avoiding excessive fatigue, overtraining and injuries. For a successful design of such programs, it is important to use integrated training monitoring tools that allow recording variables representing changes in whole-body functional state, rather than fluctuations of very specific/isolated markers. One marker of the functional status of an organism, that gives an indication of the balance between the sympathetic and parasympathetic nervous system, is heart rate variability (HRV). This indicator has recently become quite popular and has been associated with numerous conditions ranging from sleep quality to sports performance [2,3,4,5,6]. 

HRV represents fluctuations in electrocardiogram (ECG) R–R time interval and reflects cardiac regulation by the autonomic nervous system based on the instantaneous sum effects of sympathetic and parasympathetic systems in response to both physical and psychological stimuli [7]. Exercise activates the sympathetic nervous system, causing an increase in myocardial contractility and vasoconstriction of peripheral blood vessels, which are moderated by the parasympathetic nervous system [8,9]. Recent reviews suggest that HRV biofeedback is a method that is effective and safe, and is easy to learn and apply to improve sport performance [4,8]. It has also been highlighted that HRV may reflect the training-induced level of stress and recovery, which has encouraged regular HRV monitoring [3,10]. However, it has also been shown that overload training leading to overreaching has little effect on resting HRV or increased postexercise HRV, and that day-to-day load variability was not related to HRV (for review, see Bellinger et al., [3]). Thus, although HRV has been investigated extensively, its practical use in everyday training remains controversial [2,3].

Handling individual training-induced responses is particularly relevant in such sports as swimming, where the risk of overreaching and overtraining is high because of the very large volume and monotonous training loads and early specialization by athletes [11]. As reviewed by Koenig et al. [12], although HRV monitoring in swimming may have some beneficial outcomes, it is also recognized that there is a lack of translational approaches to apply the current evidence to general practice. Several recent studies demonstrated the relationships between the autonomic nervous system, performance, and fatigue, showing that HRV responses appeared to depend on the training status, age, and environmental factors of the athletes [13,14,15,16,17]. However, the vastly different populations and contexts of these studies create uncertainty about the interpretation of their findings, which may partly explain why sport practitioners are still reluctant to incorporate HRV monitoring into their arsenal of training tools.

Because of the above considerations, we feel that the utility of HRV as a training load monitoring tool requires further examination in young competitive swimmers. Adolescents were selected for the investigation because they may be particularly vulnerable to overreaching because of their structural, biomechanical, and, consequently, functional alterations related to the intrinsic effects of rapid somatic growth and development [18,19]. Adolescence is also associated with increased sensitivity to environmental conditions (stress related to school activities, relationships with parents, friends, etc.) and therefore it is particularly important to control stress and recovery in competitive athletes of this age. Better understanding of training-specific HRV responses may optimize athletic activity and prevent the development of long-lasting fatigue. The main aim of this study was therefore to analyze HRV associations with training load and sleep duration during the 11-week training macrocycle of young swimmers. We hypothesized that HRV measurements would be feasible during the preparatory training period of young swimmers, that total training volume and high-intensity (HI) training volume would be inversely correlated with HRV, and that sleep duration would be directly correlated with HRV.

## 2. Materials and Methods 

### 2.1. Participants

Twenty-five adolescent swimmers at national level from the same swimming school were initially recruited to the study. The inclusion criteria were: (1) healthy, (2) absence of any clinical history of neuromuscular disorders within preceding year, (3) regular swimming training of at least 5 years; (4) competitive at least at national level, and (5) early or middle adolescence (13–16 years). However, three athletes were excluded because of inconsistent R–R interval measurements. The characteristics of the participants for whom data were analyzed (n = 22) are shown in Table 1. Athletes were following periodized training programs that were individually developed and prescribed by their swimming coach with the main intention of preparing them for the national swimming championships. This period of training had been preceded by a nonathletic period of passive rest during the summer holiday and a transition period from rest to regular training that comprised light training 4–5 times per week for two weeks. A standard training week comprised 5–7 swimming sessions, most of which were held in the afternoons on Mondays to Fridays (the days they all attended lessons in the school during the morning hours), and some sessions in the mornings during the week. Just before each afternoon swimming session, participants performed dry-land mobility and strength exercises of 30–45 min in duration. Training required a minimum of 8 hours per week. There was a substantial variation in training compliance (76.0 ± 17.2%), but the athletes with low compliance (two below 50%) were not excluded from the analyses because load variability was considered as a factor in HRV. Swimmers specialized in events of different strokes at short to middle distances and had at least 5 years of swimming training experience. All participants and their parents signed an informed consent form prior to participation. The study was approved by the relevant bioethics committee of the Lithuanian Sports University (No. BEK-KIN(B)-2019-184). The study was conducted in accordance with the Declaration of Helsinki.

### 2.2. Study Design

The swimmers were followed for 77 days of the preseason training period (September to November) of which 11 days were preplanned to be without training sessions. HRV and sleep duration were measured daily by the athletes themselves. The intensity and volume of the training load of each swimmer were recorded daily by the swimming coaches in the athlete’s training log and then provided to the researchers. The training loads were not adjusted or corrected as a result of the data collected in the framework of the current study, and decisions to select training loads, modalities, intensities etc., were completely at the discretion of the athletes’ coaches. During the period of the study, the swimmers took part in 2–3 local competitions. One week before the experimental period, participants were familiarized with the use and proper fitting of heart rate and sleep monitoring devices, their somatic maturational status was verified by experienced medical staff using the Tanner scale [20]. We evaluated participants’ body height (Anthropometry Martin, GPM Siber-Hegner, Geneva, Switzerland), body weight and fat percentage (Tanita, model TBF 300; Tokyo, Japan), jump height (Power Timer Testing System, Newest, Finland), dominant leg knee extension strength at angular speed 30 °∙s^−1^ (System 3; Biodex Medical Systems, Shirley, NY, USA), and VO_2peak_ on a stationary cycling ergometer (Ergoline, Windhagen, Germany) using a portable breath-by-breath analyzer (Oxygen Mobile; Jaeger/VIASYS Healthcare, Hoechberg, Germany).

### 2.3. Training Load Monitoring

The specific swimming training was performed in a 25-m standard swimming pool and was stratified according to heart rate (HR) into intensity zones 1 to 4: <139 beats/min (bpm) for Zone 1, 150 ± 10 bpm for Zone 2, 170 ± 10 bpm for Zone 3, and >181 bpm for Zone 4; sprints with maximal exertion level were classified as Zone 5. The intensity control was based on criterion speed individually directed by coach in conjunction with pulse count using a chronometer “Alpha” (Sport-Thieme, Grasleben, Germany) for 15 s after distinct swimming tasks. All swimmers were experienced in manually measuring pulse. Swimming at Zones 4 and 5 was considered HI training. Total swimming volume and HI swimming volume were analyzed as distance covered during the training period.

### 2.4. HRV Measurement

Over the 11 consecutive weeks of their preparatory training period, swimmers daily measured their HRV in the supine position for 2 min immediately after awakening in the morning by positioning an H10 Bluetooth HR strap (Polar Electro, Kempele, Finland) paired with a freely available smartphone application (Elite HRV, Ashville, NC, USA) on their chest. This system has been used previously for daily measurement of HRV [21]. We analyzed the square root of the mean sum of the squared differences between R–R intervals (RMSSD), which was converted by logarithmic transformation (lnRMSSD) to avoid outliers and simplify the analyses, as suggested by Nakamura et al. [22]. Data files were visually inspected for artefacts, and corrections made manually if necessary. Both RMSSD and lnRMSSD are recognized markers of parasympathetic activity and are the preferred HRV markers for field-based monitoring [2].

### 2.5. Sleep Monitoring

During the study, all participants slept in their homes and went to bed at around 11 pm. Participants wore an activity monitoring bracelet on the wrist of their nondominant hand every night. Daily sleep duration was monitored by a commercially available wrist-worn sleep/activity tracker, Mi band 2 [23]. Moderate intraclass correlation coefficients (ICC) of 0.62–0.75 have been reported for the Mi Band 2 sleep duration assessment [24].

### 2.6. Statistical Analyses

All variables are expressed in terms of mean ± standard deviation (SD). Levene’s test was used to test the homogeneity of variances. The Kolmogorov–Smirnov test was used for checking distribution normality. A one-way analysis of variance (ANOVA) was used to determine the effects of time on dependent values (load, HI load, sleep, RMSSD and lmRMSSD) across weeks or days during the training period. If a significant main effect was found, the significance of the difference between means was estimated by applying paired *t*-tests. Statistical power (observed power, OP) was calculated and presented where appropriate. During the monitoring period, there were 23 cases when individual swimmers had 3–5 consecutive days of high total training volume (>6 km/day), and 24 cases of 3–5 consecutive days of passive rest not related to injuries or illnesses (on several occasions for some individuals). These cases were analyzed separately by comparing HRV at the beginning and end of each period using paired *t*-tests. An independent *t*-test was used for comparisons of baseline values between females and males. The level of significance was set at 5% (*p* < 0.05). Pearson’s correlation (*r*) was used to quantify the relationship between each daily RMSSD or lnRMSSD and the previous day’s training variables. The correlation was considered strong if *r* > 0.5; moderate if *r* = 0.3–0.5; and weak if *r* = 0.1–0.3 [25]. All data analyses were performed using IBM SPSS Statistics software (v.22; IBM Corp., Armonk, NY, USA).

## 3. Results

Body weight, height, and dry-land performance were higher in males than in females and fat percentage was lower in males than in females (both *p* < 0.05) (Table 1). Because HRV and maturation level were not gender dependent, all the data were pooled for the analysis.

### 3.1. Training Volume

**Figure 1:**
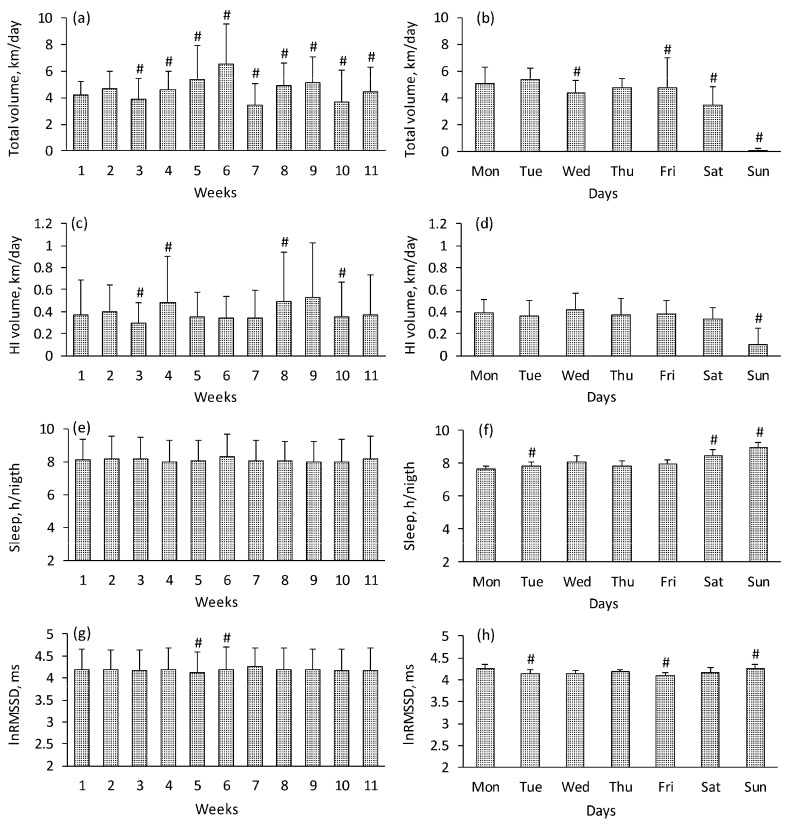
Total (**a**,**b**) and high-intensity (**c**,**d**) training volume, sleep (**e**,**f**) and lnRMSSD (**g**,**h**) in swimmers across the training period (average ± SD). Note: # *p* < 0.05 vs. previous value.

Total swimming training volume was 232.1 ± 81.7 km during the 11 weeks, with training at HI (combined zones 4 and 5) comprising 6.7 ± 4% of total swimming volume. There was a significant week-by-week variation in total swimming volume, with the highest peak at week 6 (>6.0 km/per day, *p* < 0.05, OP = 0.99) (Figure 1). Peaks of the swimming volume that reached HI zones were evident in weeks 4, 8, and 9 (about 0.5 km/per day, *p* < 0.05, OP = 0.65). Total training volume was reduced on Wednesdays (*p* < 0.05 compared with Mondays and Tuesdays, OP = 0.95) and Saturdays (*p* < 0.05 compared with any other day, OP = 0.99), with usually no load on Sundays. HI swimming volume did not vary significantly between days within a week.

### 3.2. Sleep

Average duration of sleep ranged from 7.32 ± 1.04 to 8.73 ± 0.69 h per day between subjects, with no change in weekly sleep duration over the training period (Figure 1). Within a week, swimmers were getting less sleep on Mondays compared with any other day (*p* < 0.05 in all cases, OP = 0.99) and particularly compared with Sundays (>1 h, *p* < 0.05).

### 3.3. Resting HRV (lnRMSSD)

During the training period, the group average values of HR, RMSSD, and lnRMSSD were 68.6 ± 6.9 bpm, 73.0 ± 24.7 ms, and 4.23 ± 0.35 ms, respectively. Weekly variation in resting lnRMSSD reached significance in weeks 5 and 6 (*p* < 0.05 compared with the previous week) (Figure 1) but was generally minimal and usually did not coincide with training volume peaks. Interestingly, morning lnRMSSD showed a progressive decrease within each week from a peak on Sundays and Mondays to the lowest values on Fridays (*p* < 0.05, OP = 0.98). In addition, lnRMSSD decreased from 4.52 ± 1.91 to 4.32 ± 1.73 ms after 3–5 days of high total training volume (> 6 km/day), and increased from 4.09 ± 1.63 to 4.29 ± 1.54 ms after 3–5 days of rest (*p* < 0.05 in both cases).

### 3.4. Correlations between Daily HRV, Training Volume, and Sleep Quantity

**Figure 2:**
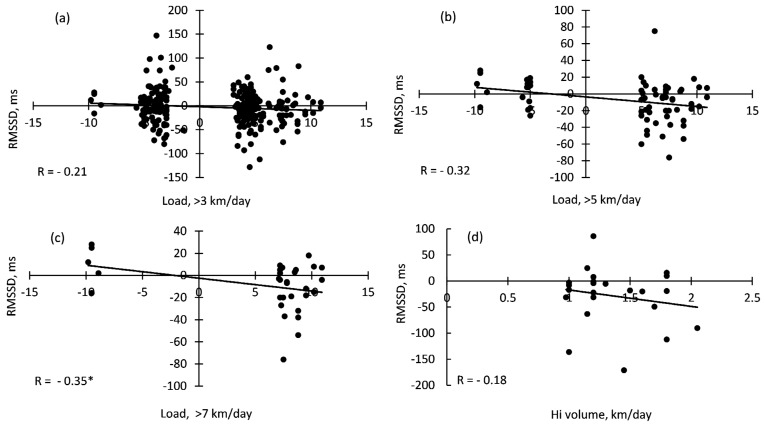
Correlations between individual values of RMSSD shifts and total (**a**,**b**,**c**) or high-intensity (HI, d) training volume shifts. Note: * *p* < 0.05.

Individual day-to-day lnRMSSD values varied up to 50% across the training period (*p* < 0.05) (Table 2). There was a weak inverse correlation (*r* > −0.10) of lnRMSSD with total swimming volume for 14 of the 22 subjects, which reached significance in only five swimmers (*r* > −0.27, *p* < 0.05). The correlations of lnRMSSD day-to-day variability with HI training volume or sleep volume were even more trivial and unpredictably shifted from positive to inverse depending on the subject. A moderate inverse relationship was detected between lnRMSSD and large shifts in total training volume (*r* = −0.35, *p* < 0.05 in cases of a >7.0 km/day swimming volume increase/decrease) (Figure 2). No significant correlations were detected between lnRMSSD and small or moderate fluctuations (increase/decrease by >3.0 or >5.0 km/day) in total volume or HI training volume.

## 4. Discussion

The main finding of the study was a quite consistent reduction in HRV in response to markedly increased training load such as a sharp increase in training volume or maintenance of high training volume for 3–5 consecutive days. By contrast, day-to-day HRV in general reflected poorly swimming training volume or sleep quantity, although this was also highly individual. These findings suggest that measurement of morning HRV under home-based conditions could be included as an indicator of the balance between the magnitude of the physical load and the young athlete’s tolerance capacity within the intensified training periods.

The data obtained in the present study support the current understanding that HRV monitoring does not allow the separation and classification of different subcategories of stress, but may be useful in helping the practitioner to recognize the overall fatigue level [26,27]. It is quite well established that one of the most popular HRV indexes, lnRMSSD, decreases considerably in response to extreme loading, reflecting decreased vagal (parasympathetic) heart control, and this is often associated with athlete fatigue and impaired performance [2,7,16,28,29,30,31]. Our study found that during the days when training volume was high, lnRMSSD on the following morning was lower than weekend values. Bearing in mind that our subjects were young but otherwise well-trained swimmers who were expected to cope well with individual programs adapted to their age, gender, training history, and ability level, the data suggest that observed fluctuations in HRV are of practical value in sports training. Similar results have been observed in a number of studies that required extended cardiac demands in response to endurance exercise training [16,29,32]. For instance, alterations in resting autonomic function as reflected by changed HRV have been associated with functional overreaching in triathletes [33] or injury incidence in swimmers [15].

In the setting of the current study, it was expected that day-to-day HRV monitoring would help detect stress and recovery levels in young swimmers over the preparatory training period of 11 weeks. With this information in hand, coaches would be able to more precisely prescribe the training loads for each workout according to the athlete’s condition in a given timeframe and hence better prevent young athletes from excessive fatigue and insufficient recovery, leading to overloading, overreaching, overtraining, and associated negative outcomes such as staleness, injuries, or lack of progress. There was no significant instability of resting HRV in any of the subjects during the whole monitoring period, indicating that excessive training loads had been avoided. However, for the optimization of progress in performance, sufficient training loads are required during at least some of the weekly training sessions (as after several consecutive days of intensified training, or HI training), and thus it could be speculated that some degree of the change in resting HRV should be desirable as indicating the triggering of beneficial adaptations. On a group level, daily lnRMSSD variations did not correlate well with the previous day’s load and there was frequently inconsistent shifting of lnRMSSD in the direction opposite to that expected. Individual changes were more predictable, but still the change was significant in only five of the 22 subjects (<25%). Possible reasons for such discrepancies between the individual and group level analyses have been described comprehensively elsewhere [34,35], and include large interindividual differences between athletes in HRV parameters at baseline, HRV changes during training, age, gender, perceived efforts, and anxiety. Of interest, we noted a tendency of daily HRV variability to better reflect training load in the adolescents with low adherence to training and low total swimming load in general; in addition, greater vagal activity was associated with smaller fluctuations lnRMSSD across training days.

It was rather unexpected that values of day-to-day HRV (lnRMSSD) did not correlate with the duration of night sleep. Sleep is indispensable for recovery and performance progress in athletes [36,37]. It is well established that the autonomic nervous system plays an important role in the modulation of cardiovascular functions during the onset of sleep and in transition between sleep phases [38]. However, variation in the duration of sleep did not emerge as a significant influence on regulation of the autonomic nervous system in the current study, which included no external manipulation of sleeping patterns. However, it could hypothetically be that the association was masked because of the timing of HRV measurement, which occurred not during the night sleep but only for 2 min after the morning awakening. It is also possible that HRV is more sensitive to sleep quality and consistency rather than the total duration of sleep [6], but analysis of this was outside the scope of the current study.

For the sake of simplicity, we used easily accessible devices and limited HRV analyses to the single parameter of RMSSD, which was transformed to lnRMSSD. Measuring RMSSD alone might not give a full picture of the mechanisms underlying changes in cardiac autonomic regulation [8,27,39]. However, we consider that simplicity of monitoring and its interpretation are essential to encourage usage of HRV measurement as a valid tool in sports practice. HRV recording is relatively straightforward because numerous free applications have been developed for regular smartphones [4]. In addition, although the recorded HRV data could be available almost instantaneously to the coach or other staff of the sports team for inspection, analysis, and feedback, the interpretation of HRV and its changes is not simple because response magnitude and timing are individually determined by multiple factors including biological variation, emotions, and training context [2,27,40,41,42]. 

### 4.1. Limitations

Several limitations of the current study should be acknowledged. Although the subjects participated in several sessions devoted to learning the HRV recording procedure and a one-week familiarization period, the data from which were not included in the analysis, it was not possible to monitor the HRV measurement procedure during the study to ensure that it had always been conducted correctly. However, the familiar home environment and absence of the researchers allowed the procedure to be at least well standardized and to obtain data not affected by additional stressors. It could well be that timing of the HRV measurements did not allow estimation of the real impact of the training loads on HRV, because the training sessions were typically half a day apart from the HRV recordings; i.e., subjects might have either fully or partially recovered from the most recent training session after the night’s rest (with additional recuperation until the next workout), or they had accumulated additional stress during the day up to training. These aspects might have diminished the reported associations between training loads and HRV.

Circadian rhythms have also been linked to HRV [43,44]. The subjects in the current study, all schoolchildren, were waking up and measuring HRV in the morning at quite consistent times during the week (except in some rare cases when they had training sessions early in the morning) but later on weekends. Both of these factors possibly contributed to variability or bias of the recorded data, with the longer sleep duration during weekends affecting HRV not only because of the longer rest, but also because of the later time of the measurement, and the more relaxed approach when there were no school activities or training sessions planned.

We neither controlled nor recorded the subjects’ diet, fluid, and food supplementation but the HRV measurements were always conducted in the fasted state just after awakening, which mitigates the possible effects of fluctuations in dietary intake during the previous day. Finally, biochemical measures of stress such as cortisol to testosterone ratios would possibly have been useful in the interpretation of the relationship of HRV with training loads and recovery [45].

### 4.2. Practical Application and Recommendations

Morning HRV could be consistently and reliably recorded on a daily basis by young (teenage schoolchildren) competitive athletes in their home environment by means of commercially available monitors and without supervision from parents, coaches or researchers. Morning HRV appears to be a potential marker of the internal training load during the intensified training of young swimmers. However, morning HRV weakly reflected the day-to-day fluctuations of training volume or intensity. Therefore, simultaneous collection of additional subjective self-reported data such as perceived exertion of each of the training sessions, as well as perceived sleep quality, amount of stress, muscle fatigue and soreness by using customized questionnaires on a daily basis could be recommended for the more thorough evaluation of the changes in athletes’ wellness [46] which could be of use in making decisions for the adjustments in training loads. More demanding training load monitoring tools, such as biochemical markers [47,48,49], could be considered to be included into the training monitoring. 

## 5. Conclusions

This study showed a quite consistent reduction in HRV in response to markedly increased training loads for several consecutive days. Although highly individual, day-to-day fluctuations in the morning HRV was not consistently associated with swimming training volume or sleep quantity. Taken together, these findings suggest that HRV fluctuations have limited value to quantify the balance between the magnitude of the physical load and young athletes’ tolerance capacity, although under sharply increased training load or extended periods of high volume training conditions, it may play a relatively important role.

## Figures and Tables

**Table 1:** Characteristics of the participants (mean and standard deviation).

Variable	Male (n = 7)	Female (n = 15)	Total (n = 22)
Age, years	15.4 (0.7)	13.8 (0.6) ^	14.3 (1.0)
Height, cm	179.5 (6.0)	165.1 (6.7) ^	169.7 (9.3)
Weight, kg	65.5 (6.7)	56.5 (6.1) ^	59.4 (7.5)
Body fat, %	10.8 (3.9)	21.5 (3.8) ^	18.1 (6.3)
Knee extension peak torque, Nm/s	197.3 (20.7)	141.4 (27.5) ^	159.2 (36.6)
Vertical jump height, cm	40.2 (2.1)	31.2 (3.3) ^	33.8 (5.1)
VO_2_peak, mL/min/kg	47.8 (5.0)	38.8 (6.8) ^	41.6 (7.5)
Maturity			
Tanner II, n (%)	1 (14.3 %)	2 (13.3 %)	3 (13.6 %)
Tanner III, n (%)	5 (71.4 %)	11 (73.4 %)	16 (72.8 %)
Tanner IV, n (%)	1 (14.3 %)	2 (13.3 %)	3 (13.6 %)

Note: ^ *p* < 0.05 vs. male.

**Table 2:** Individual values of R–R intervals (RMSSD) converted by logarithmic transformation (lnRMSSD) across the training period and correlations (Correl) with total and high-intensity (HI) training and sleep duration.

LnRMSDD, ms							Correl LnRMSDD		
No	Gender	Average	Max	Min	SD	CV%	Training Volume	HI Volume	Sleep
1	M	4.30	4.91	3.61	0.29	6.7	−0.05	−0.02	−0.01
2	M	4.25	4.88	2.20	0.36	8.5	−0.22	−0.24	−0.13
3	M	4.53	4.99	4.04	0.21	4.6	−0.07	−0.03	−0.13
4	M	3.90	5.19	3.18	0.44	11.3	−0.05	0.28	−0.03
5	M	4.61	5.08	4.13	0.20	4.3	−0.31 ^#^	0.11	−0.02
6	M	4.47	5.24	3.71	0.35	7.8	−0.18	0.05	−0.08
7	M	4.18	4.92	3.78	0.25	6.0	−0.09	0.11	−0.22
8	F	4.62	5.05	3.91	0.24	5.2	−0.08	0.30	−0.17
9	F	4.12	4.85	3.00	0.37	9.0	−0.28 ^#^	0.04	−0.06
10	F	3.60	4.43	2.30	0.44	12.2	−0.20	−0.01	0.00
11	F	3.38	4.29	2.40	0.41	12.1	−0.22	−0.12	0.06
12	F	3.65	3.99	3.22	0.18	4.9	−0.20	−0.06	−0.08
13	F	4.26	5.00	3.26	0.31	7.3	−0.05	0.25	−0.27
14	F	3.80	5.06	2.48	0.59	15.5	−0.17	0.23	0.41 ^#^
15	F	4.84	5.32	4.20	0.23	4.8	−0.03	−0.21	−0.04
16	F	4.21	4.69	3.56	0.23	5.5	−0.06	−0.10	−0.16
17	F	4.59	5.13	3.71	0.36	7.8	−0.16	0.15	−0.05
18	F	4.03	5.00	3.26	0.38	9.4	−0.27 ^#^	0.02	−0.08
19	F	4.19	4.68	3.09	0.29	6.9	−0.29 ^#^	0.26	0.30 ^#^
20	F	3.95	4.89	2.83	0.55	13.9	−0.45 ^#^	0.03	−0.26
21	F	4.23	4.90	3.4	0.35	8.3	−0.16	0.21	−0.04
22	F	4.01	5.32	3.18	0.37	9.2	−0.17	−0.03	0.21
Mean		4.17	4.90	3.29	0.34	8.1	−0.17	0.06	−0.04
SD		0.37	0.33	0.59	0.11	3.1			

Note: M, male; F, female; SD, standard deviation; CV, coefficient of variation; HI, high-intensity training volume; ^#^ *p* < 0.05.
